# Suitability of various chromatographic and spectroscopic techniques for analysis and kinetic degradation study of trelagliptin

**DOI:** 10.1038/s41598-017-17642-1

**Published:** 2017-12-08

**Authors:** Wafaa A. Zaghary, Shereen Mowaka, Mostafa A. Hassan, Bassam M. Ayoub

**Affiliations:** 1 Pharmaceutical Chemistry Department, Faculty of Pharmacy, Helwan University, Ein Helwan, Cairo 11795 Egypt; 2 Analytical Chemistry Department, Faculty of Pharmacy, Helwan University, Ein Helwan, Cairo 11795 Egypt; 3 Pharmaceutical Chemistry Department, Faculty of Pharmacy, The British University in Egypt, El-Sherouk city, Cairo 11837 Egypt; 4 The Center for Drug Research and Development (CDRD), Faculty of Pharmacy, The British University in Egypt, El-Sherouk city, Cairo 11837 Egypt

## Abstract

Multifaceted comparative analytical methods for trelagliptin (TRL) were investigated, applied to ZAFATEK tablets and HPLC-UV was selected for a degradation kinetic study. UPLC-MS/MS (Method I), UPLC-UV (Method II), HPLC-UV (Method III), UHPLC-UV (Method IV) and direct UV (Method V) methods were developed. Methods (I-V) showed satisfactory results using TRL concentration ranges of 50–800 ng/mL, 2.5–80 μg/mL, 5–100 μg/mL, 5–100 μg/mL and 5–50 μg/mL, respectively. Multiple Reaction Monitoring (MRM) of the transition pairs of m/z 358.176 to 134.127 for TRL and m/z 340.18 to 116.08 for alogliptin (IS) were employed utilizing positive mode Electrospray Ionization (ESI). The degradation kinetic study (Method VI) was carried out using 1 N HCl based on three different temperatures (70 °C, 80 °C and 90 °C). Through the optimized method-3, a good chromatographic separation of TRL from its major degradation product was achieved. Arrhenius plot was used in the kinetic study and the apparent 1^st^ order degradation rate constant (K), t_1/2_, t_90_, and the activation energies were calculated for each temperature and at 25 °C. The optimized UPLC-MS/MS method is suitable for further TRL assay either in biological fluids or in the presence of impurities.

## Introduction

Trelagliptin (TRL) is a new antidiabetic drug that administered as a monotherapy for the treatment of diabetes mellitus (type II) and classified as one of dipeptidyl peptidase-4 (DPP-4) inhibitors. Its mechanism of action involves rising of the endogenous glucagon like peptide-1 (GLP-1) and other hormone levels by inhibiting the degrading enzyme DPP-4^1,2^. TRL administration once weekly showed high efficacy and good safety profile^1,2^. Pharmacokinetic study of TRL was performed in rat plasma^3^ using LC-MS/MS, HPLC method was reported for quantification of the enantiomeric purity for TRL with UV detection at 260 nm^4^ and another HPLC-UV method was reported for the analysis of impurities that yielded from TRL synthesis process^5^.

In the present work, multifaceted comparative analysis for trelagliptin (TRL) was investigated and applied to ZAFATEK tablets using UPLC-MS/MS on AGILENT SB- C_18_ column, UPLC-UV on HYPERSIL Gold C_18_ column, HPLC-UV on BDS HYPERSIL C_18_ column, UHPLC-UV on SYMMETRY C_18_ column besides direct UV methods. A validated HPLC-UV method was selected for a degradation kinetic study due to better separation for the degradation products and lower cost than LC-MS/MS method. Up to our knowledge, no methods were found in literature dealing with TRL kinetic degradation study. Consequently, the innovation of the underlying study is based on the multifaceted analysis for TRL in addition to a detailed kinetic degradation study. Apparent 1^st^ order degradation rate constant (K), t_1/2_, t_90_, and the activation energies were calculated at different temperatures and at 25 °C.

**Figure 1:**
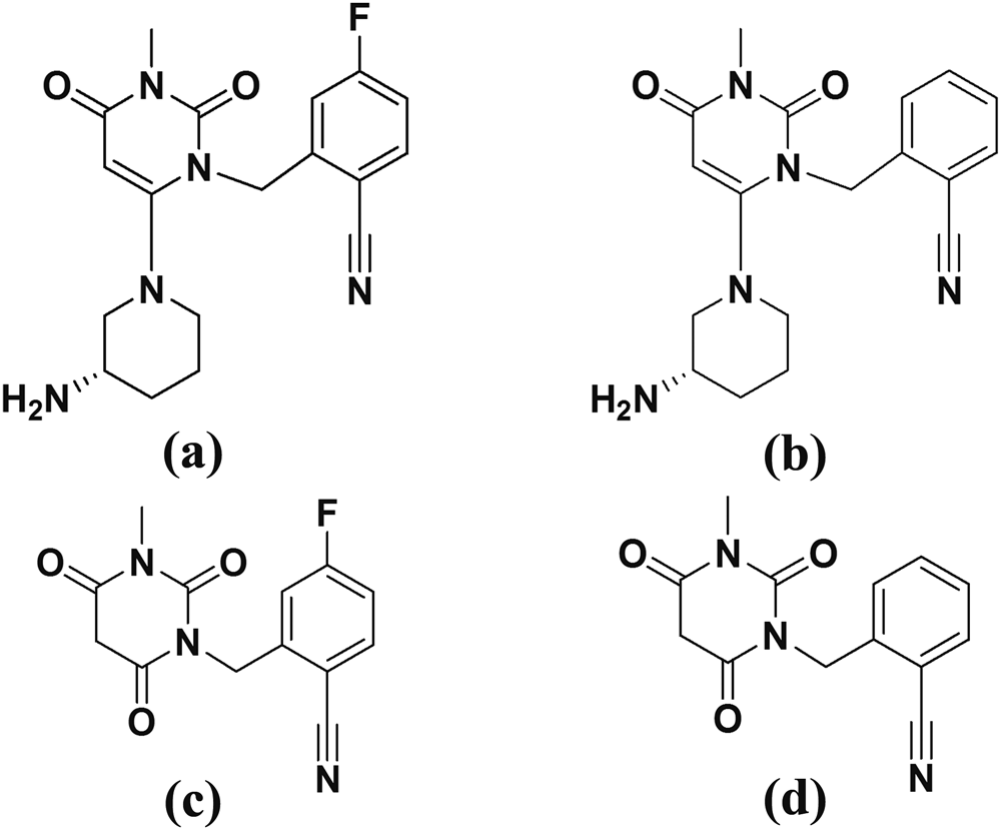
Chemical structures of TRL (**a**), ALO (**b**), TRL main degradation product (**c**) and ALO main degradation product (**d**).

The acid degradation product of TRL in literature was found to be “2-[(3-methyl-2,4,6-oxo-tetrahydro-pyrimidin-1 (2 H) yl)-methyl]-4-fluorobenzonitrile”^5,6^. Due to structure similarity between TRL (Fig. 1a) and alogliptin (ALO) (Fig. 1b), the results of a previously developed forced degradation study of ALO^7^ was considered during the design of the current TRL degradation study. Therefore, we focused on acid degradation, as ALO was mostly sensitive to acid stress conditions. Furthermore, the alkaline degradation, 0.3% H_2_O_2_ oxidative degradation, UV light and heat stress conditions were checked for TRL with results in agreement with degradation outcomes from ALO^7^ with an identical chemical structure (Fig. 1a,b) except with a less fluorine atom. In addition, the obtained TRL degradation product (Fig. 1c) showed a similar degradation behavior to ALO in the acidic medium (Fig. 1d).

## Materials and Methods

### Instruments

WATERS UPLC system (USA), TQ detector supplemented with electrospray ionization source (USA) and Agilent SB-C_18_ column with dimensions (1.8 µm) 50 × 2.1 mm were used. Mass Lynx software version 4.1 was used. For the LC-UV methods, UPLC Ultimate 3000 supplied by Thermo Fisher (USA) was used. UPLC-UV on HYPERSIL Gold C_18_ column (1.9 μm) 50 mm × 3 mm, HPLC-UV on BDS HYPERSIL C_18_ column (3 μm) 100 mm × 3 mm and UHPLC-UV on SYMMETRY C_18_ column (2.2 μm) 100 mm × 2.1 mm were applied. In addition, a DAD-3000RS Detector (USA) and a WPS-3000TRS autosampler (Thermo scientific, USA) were used. For the UV Spectrophotometer: JASCO double-beam UV spectrophotometer (S/N C367961148, Japan) supported with SPECTRA MANAGER software was used. Degassing of the mobile phases and samples carried out using a sonicator S 60 H supplied by Elmasonic (Germany). Monitoring and adjusting the hydrogen ion concentration (pH) of the mobile phase and degradation samples via digital pH meter supplied by Jenway (UK).

### Chemicals, solvents and working solutions

TRL (99.0%) and ZAFATEK 50 mg tablets were supplied from Takeda Pharmaceuticals. Deionized water, Phosphoric acid, Formic acid, Potassium Dihydrogen Phosphate, HPLC grade acetonitrile and methanol were purchased from Sigma Aldrich (Germany). Stock solutions of TRL (1 mg/mL) and ALO (1 mg/mL) were prepared separately in methanol. Working solutions were prepared through further dilution of the stock solutions with methanol to the required concentrations. Working solutions of TRL equivalent to (10 µg/mL) and (100 µg/mL) were prepared for UPLC-MS/MS and the other multifaceted methods, respectively. Working solution of ALO (1 µg/mL) was used as an internal standard for method-I.

### Chromatographic conditions

For method I; a mixture of acetonitrile - formic acid 0.1% (80:20, *v/v*) was used as the mobile phase, filtered via a filter membrane with 0.2 µm pore size and it was degassed for 25 min. Injection volume of 7.5 µL, flow rate of 0.3 mL/min, run time for 1.5 min, cone voltage of 30 V, source temperature of 120 °C, capillary voltage of 3 KV, dwell at 0.161 sec., desolvation gas flow rate at 500 L/hr and desolvation temp. at 400 °C were implemented. Collision energy of 30 eV was used. Monitoring pairs of m/z 358.176 to 134.127 for TRL and m/z 340.18 to 116.08 for ALO (IS) in the positive mode utilizing ESI was employed.

For the proposed methods II-IV; the mobile phase was a mixture of acetonitrile – potassium dihydrogen phosphate buffer 0.05 M (50:50, *v/v*) and its pH was adjusted to 3.5 with 1 N phosphoric acid. Then, it was filtered through a filter membrane with 0.2 µm pore size and it was degassed for 25 min. The photodiode array detector, flow rate, column oven temperature and sample injection volume were adjusted to 274 nm, 0.5 mL/min, 25 °C and 10 µl, respectively.

### Sample preparation

Twenty tablets of ZAFATEK 50 mg were weighed, grinded and properly mixed in a mortar. An accurate amount that corresponds to 10 mg of TRL was transferred into a 100 mL volumetric flask and was stirred with 70 mL methanol for 30 min. Then the drug solution volume was completed with methanol and filtered to get TRL solution equivalent to 0.1 mg/mL. Aliquots of 200, 300 and 400 µL of the prepared solution were transferred into a series of 100 mL volumetric flasks and the volume was completed with methanol for method I. Two, Three and Four milliliters of the prepared solution were added to a series of 10 mL volumetric flasks and the volume was completed with methanol for the rest of methods II-V. Finally, the concentration of TRL in its pharmaceutical dosage form was calculated.

### Procedures and methods’ validation

#### Linearity

For method I; 1 mL of the working solution of ALO (IS) was added to a series of 10 mL volumetric flasks to get final ALO concentration equivalent to 100 ng/mL. Then, aliquots of the working solution of TRL were transferred to the same series of 10 mL-volumetric flasks to get TRL concentrations equivalent to 50 ng–800 ng/mL. Triplicates of 7.5 µL of each calibrator were analyzed using previously mentioned chromatographic conditions. Using six calibrators, calibration curve was obtained by plotting Peak Area Ratios (PAR) of TRL to IS, against the corresponding concentrations (C) of TRL.

Aliquots of TRL working solution were transferred separately into different series of 10 mL volumetric flasks to prepare final concentration ranges of 2.5–80 µg/mL, 5–100 µg/mL, 5–100 µg/mL and 5–50 µg/mL for methods II-V, respectively and finally 10 µL were injected in triplicates for the chromatographic methods. Using six calibrators, calibration curves were obtained for LC-UV and direct UV methods by plotting Area under the Peak (AUP) and Absorbance (A), respectively, against the corresponding concentrations (C) of TRL.

#### Accuracy and precision

TRL concentrations equivalent to (75–700 ng/mL), (7.5–70 μg/mL), (10–90 μg/mL), (10–90 μg/mL) and (7.5–45 μg/mL) were analyzed by methods I-V, respectively and finally TRL concentrations were calculated to check the accuracy of the methods with n = 5 based on recovery percent (R%). TRL concentrations (400, 500, and 600 ng/mL) for method I, (20, 25, and 30 μg/mL) for method V and (40, 50, and 60 μg/mL) for the other methods (II-IV) were assayed three times within the same day for all the multifaceted analysis methods to assess the intraday precision while interday precision was assessed for the HPLC-UV method only. Then the relative standard deviations were calculated to check the precision.

#### Assay of TRL in ZAFATEK tablets

TRL assay in ZAFATEK tablets was performed according to the procedure discussed under (linearity) and (sample preparation). TRL concentration was calculated through the corresponding regression equation. Furthermore, standard addition technique was applied to assess and ensure the validity of the proposed HPLC-UV method. Known concentrations of the pure drug (equal and ±10 µg/mL of the drug product concentration) were added to the previously determined solutions of the drug product (20, 30 and 40 µg/mL). Then the mean of the percent recoveries and standard deviation were calculated.

#### Limit of detection and limit of quantitation

Limit of detection (LOD = 3.3 * standard error of the predicted Y-value for each X in a regression) and limit of quantitation (LOQ = 10 * standard error of the predicted Y-value for each X in a regression) were calculated for all the proposed methods.

#### Robustness

Various robustness parameters of the optimized HPLC-UV (method III) were tested. Flow rate, organic strength, pH value of the mobile phase and column oven temperature were changed by ±0.02 mL/min, ±2%, ±0.1 and ±2 °C, respectively.

#### Stressed degradation conditions

According to the ICH guidelines Q1A (R2)^8^, forced degradation studies of TRL were applied. The proposed stressed conditions (acidic, alkaline, 0.3% H_2_O_2_, UV light and heat) were applied to TRL stock solutions (1 mg/mL). Acidic hydrolysis of the drug was carried out in a Fischer brand disposable tubes by mixing 2.5 mL TRL stock solution with 2.5 mL of 1 N HCl. Then the tubes were heated for different time intervals of 10, 20, 30, 40 and 50 minutes at different temperatures (70 °C, 80 °C and 90 °C). At the specified time (after cooling the tube contents), 1 N NaOH was added to neutralize the tube contents using pH meter. Alkaline hydrolysis was performed by adding 2.5 mL of TRL stock solution to 2.5 mL of 1 N NaOH and the mixture was heated at 80 °C for 2 h. After that, the mixture was cooled and neutralized with 1 N HCl using pH meter. The oxidative degradation study was conducted by adding 2.5 mL of TRL stock solution to 2.5 mL of 0.3% H_2_O_2_ then the mixture was left at room temperature for 2 h. Photolytic study was performed by exposing the TRL stock solution to UV light (4500 lx) for 2 days in a photo stability chamber. Thermal degradation study for TRL was carried out by adding 2.5 mL of the stock solution to a sealed glass vials and was kept in a water bath for 2 h with a fixed temperature 80 °C for a period of 2 h.

All the degradation samples were transferred separately into 50 mL volumetric flasks and completed to volume with a mixture of Acetonitrile (50%): Deionized water (50%). Thus, the concentration of TRL assumed to be 50 μg/mL before its exposure to any stressed conditions. Then the degradation samples were filtered through a membrane syringe filter with 0.22 µm pore size prior to its injection into the column. Finally, the samples were degassed in a sonication bath for 5 min. All samples were stored under 4 °C until analysis.

## Results

### Results obtained from methods’ development

**Figure 2:**
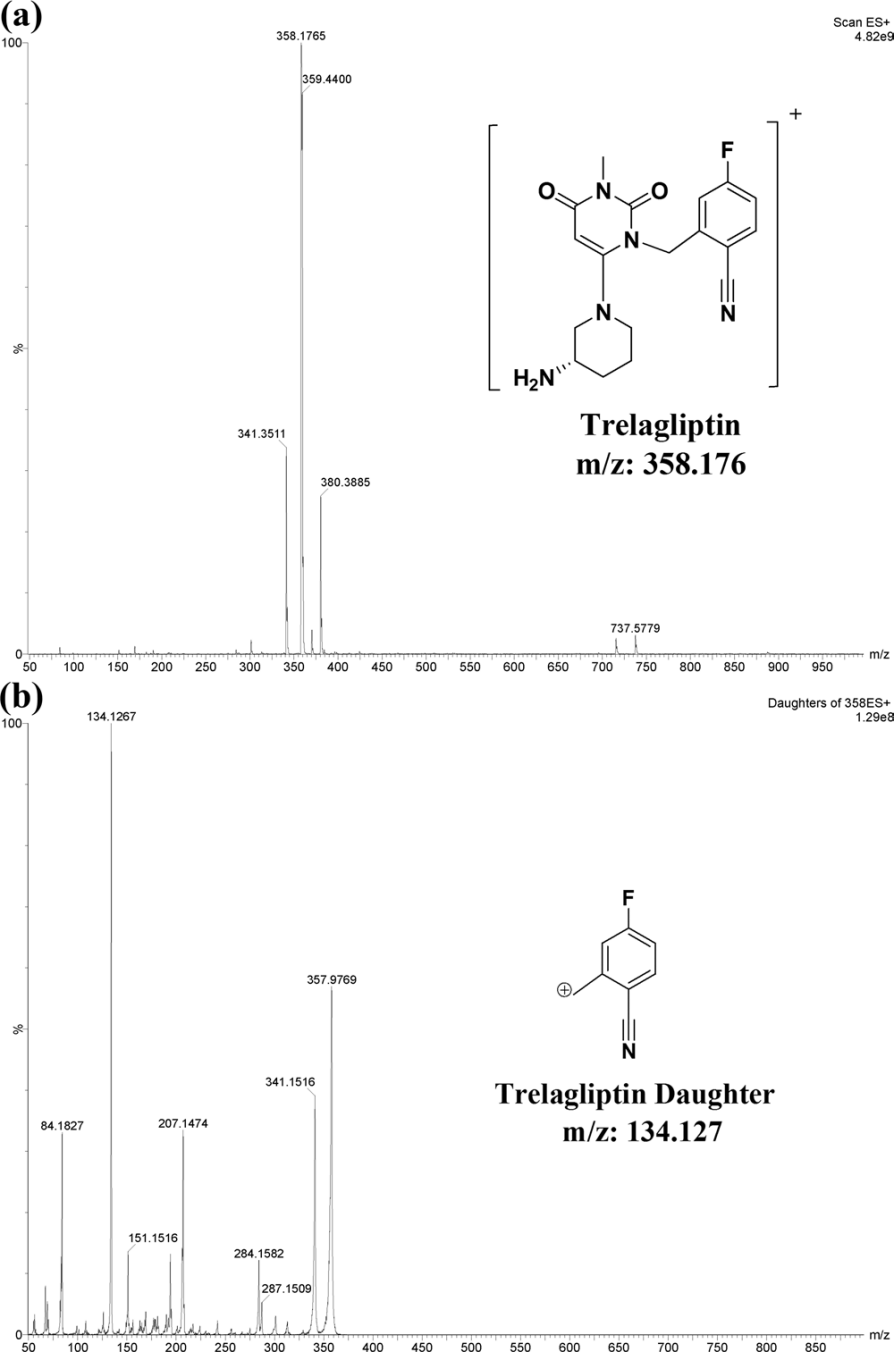
Full scan mass spectrum (**a**) and daughter ion mass spectrum (**b**) of TRL in positive ESI ion detection mode with the proposed fragment showing m/z = 358.176 & m/z = 134.127, respectively.

**Figure 3:**
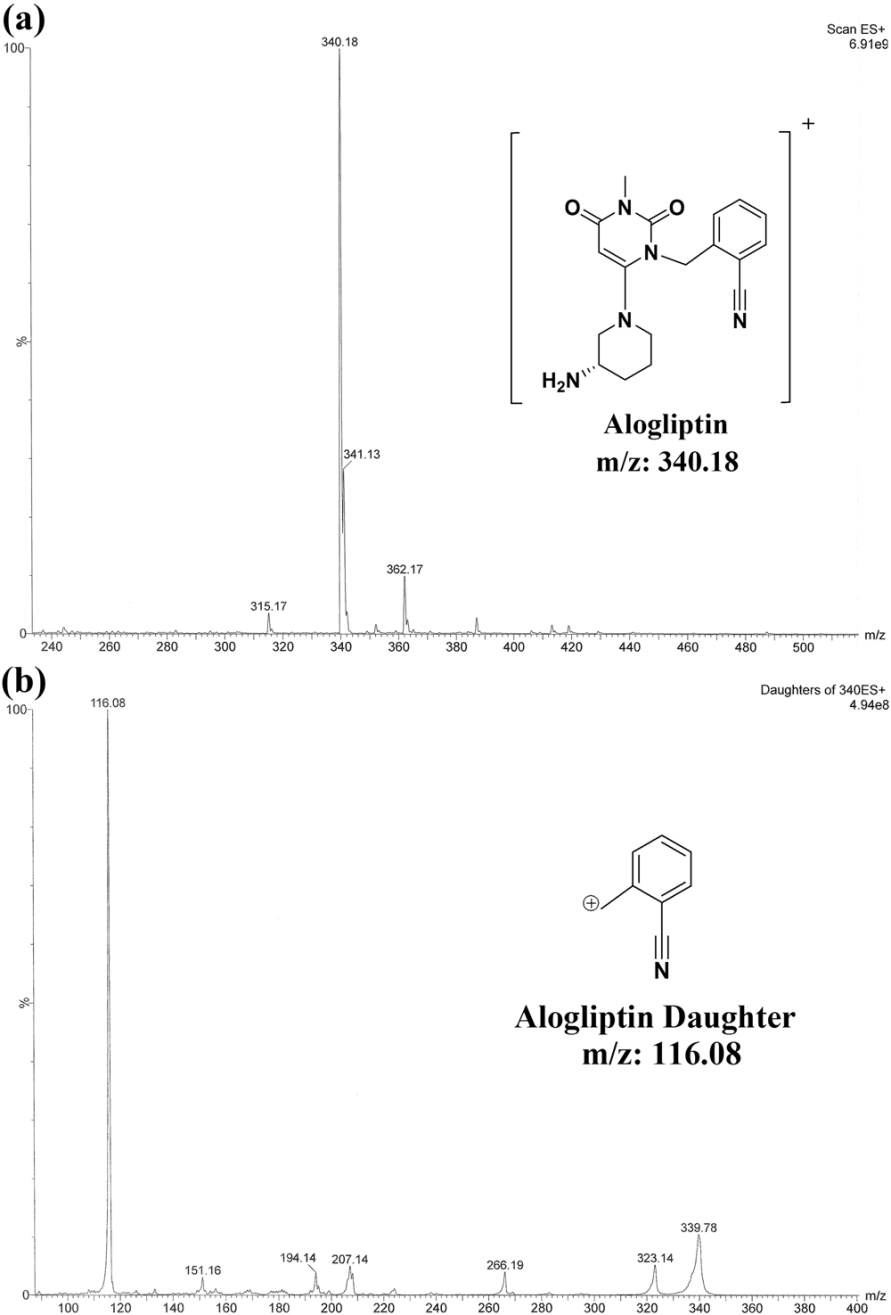
Full scan mass spectrum (**a**) and daughter ion mass spectrum (**b**) of ALO in positive ESI ion detection mode with the proposed fragment showing m/z = 340.18 & m/z = 116.08, respectively.

**Figure 4:**
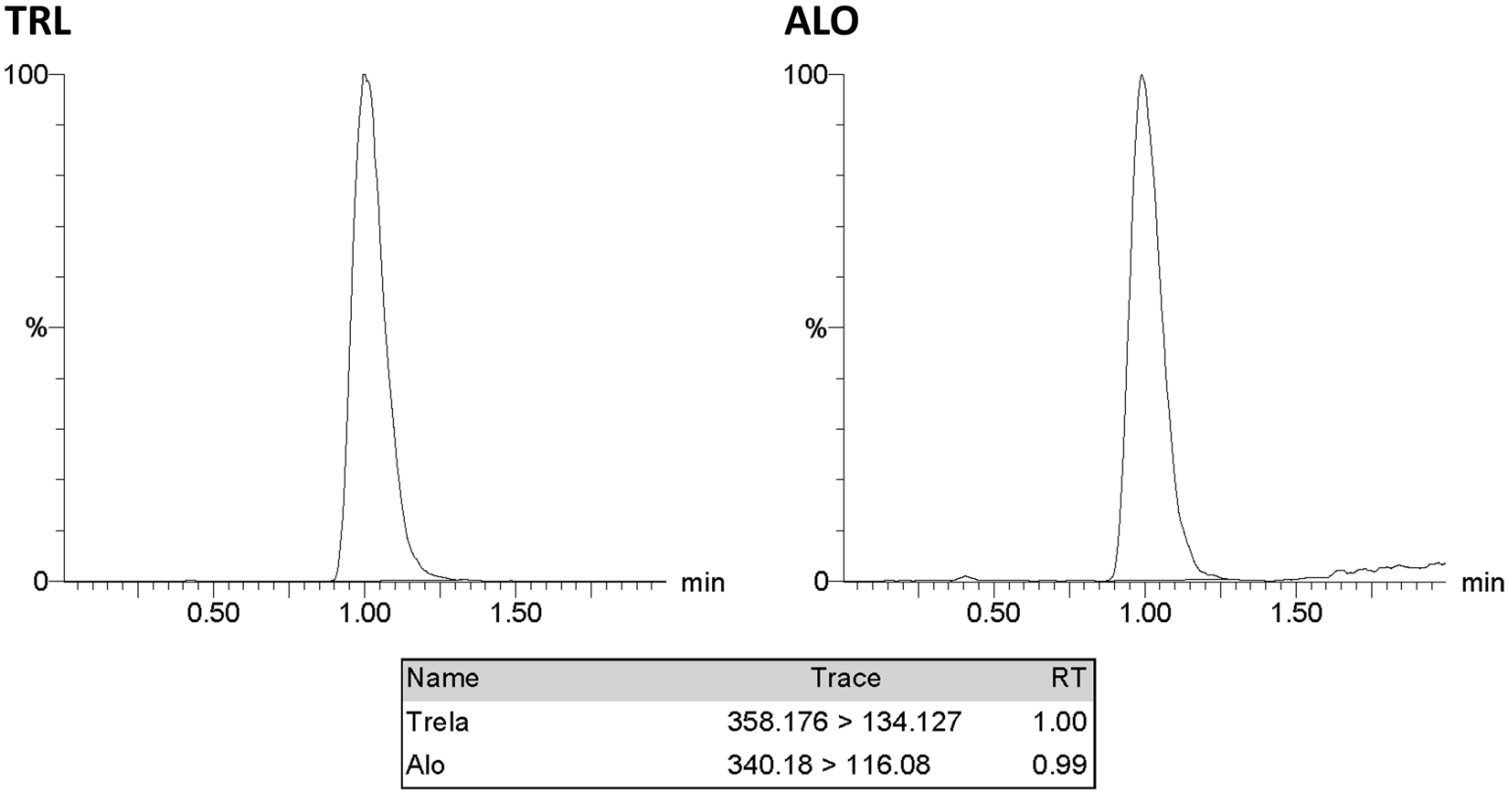
Multiple reaction monitoring (MRM) chromatogram of TRL (m/z = 358.176 to 134.127) and ALO (IS, m/z = 340.18 to 116.08).

Method I was applied successfully for TRL analysis in bulk drug and in its pharmaceutical dosage form. ALO was selected as IS because of its structural similarity to TRL. Various mobile phases (containing methanol or acetonitrile and formic acid 0.1%) were initially assessed. The best intensities were attained in the positive mode for TRL and the IS. Molecular ions of 358.176 and 340.18 were observed for TRL and ALO, respectively, on the full scan mass spectra (Figs 2a and 3a). The optimized collision energy produced daughters of 134.127 and 116.08 for TRL and ALO, respectively (Figs 2b and 3b). The MS/MS transition of 358.176 → 134.127 and 340.18 → 116.08 for TRL and the IS, respectively, were selected & resulted in optimum peak shapes after adjusting the chromatographic conditions as shown in (Fig. 4).

**Figure 5:**
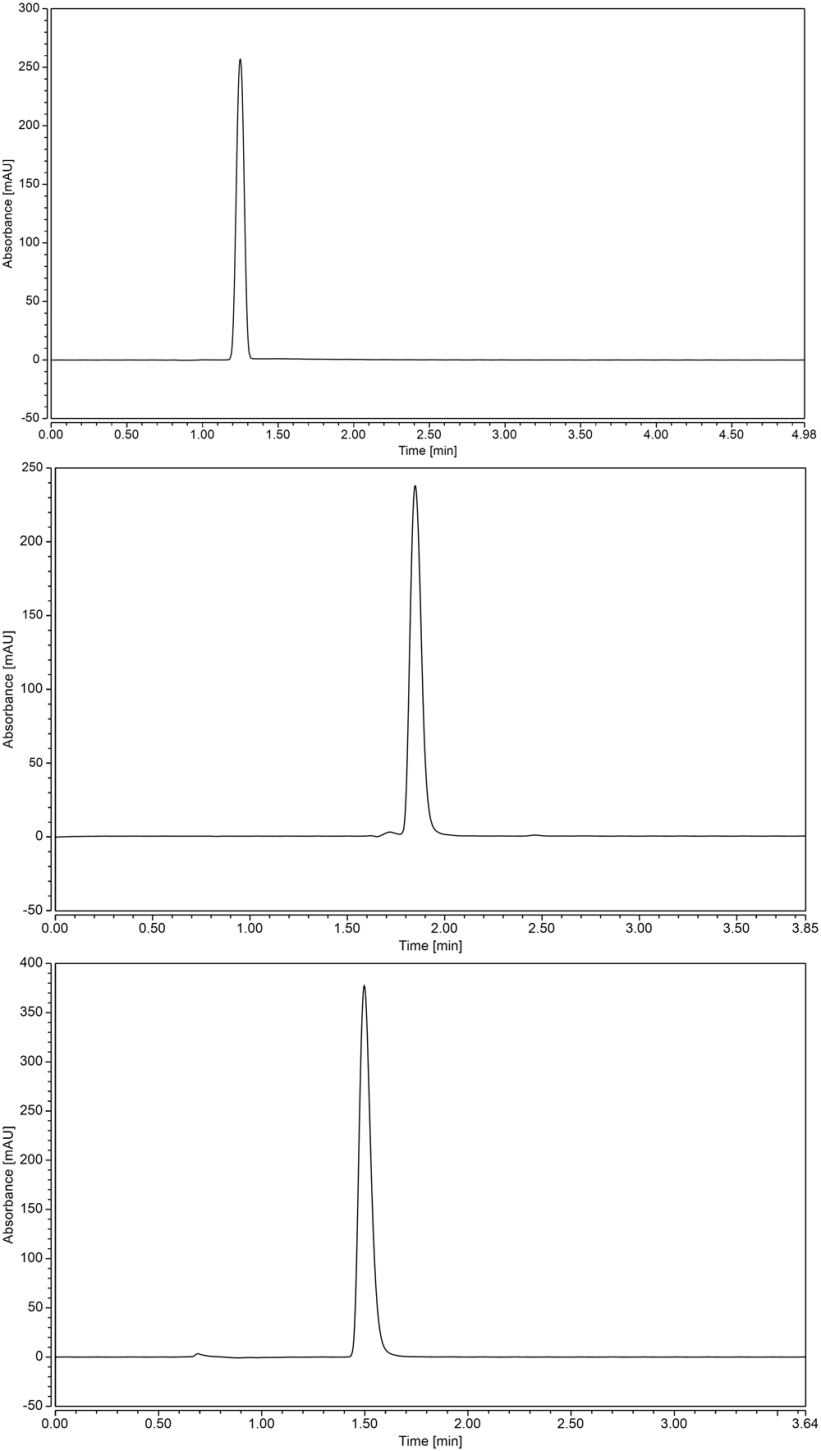
Chromatograms of TRL (50 µg/mL) using UPLC-UV (**a**), HPLC-UV (**b**) and UHPLC-UV (**c**).

**Figure 6:**
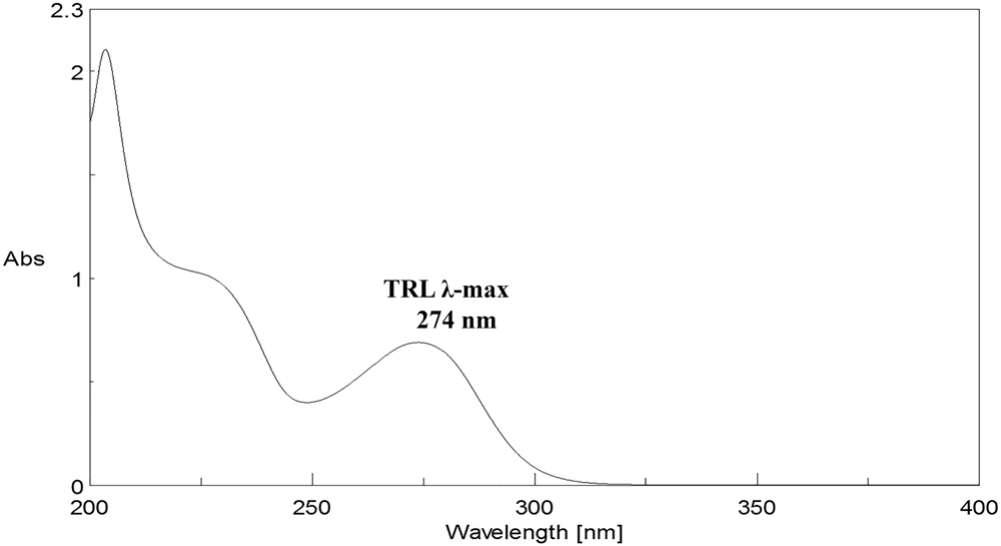
UV spectrum of TRL (25 µg/mL) showing λ-max at 274 nm.

The LC-UV best results were obtained with C_18_ columns comparable with the findings in the literature review^3,5^. The LC-UV methods were suitable for TRL analysis in bulk drug and in its pharmaceutical dosage form. Various mobile phases (containing methanol or acetonitrile and potassium dihydrogen phosphate buffer 0.05 M) and detector wavelengths (274, 245, 240 and 225 nm) were initially assessed. Phosphate buffer 0.05 M solution was found to enhance the TRL peak resolution. Optimum results for the UPLC-UV, HPLC-UV and UHPLC-UV (Fig. 5a,b and c, respectively) were obtained using a mixture of acetonitrile - potassium dihydrogen phosphate buffer 0.05 M (50:50, *v/v*) as a mobile phase. The effect of changing the pH of the mobile phase was studied by changing the pH in between the acidic region of (3–4). Optimized mobile phase of pH = 3.5 yielded good results for the three methods. The other parameters were adjusted as column temperature at 25 °C, using the injection volume of 10 µL and the flow rate of 0.3 mL/min with 2 min as run time. These conditions assisted in maintaining the chromatographic analysis under 300 bar backpressure for all methods. The optimized methods retention times of TRL were 1.26, 1.86 and 1.49 for methods II-IV, respectively. The UV absorption spectrum of TRL was recorded at λ_max_ = 274 nm using methanol as a blank (Fig. 6).

### Results obtained from the system suitability tests

The optimized HPLC-UV method was assessed for system suitability tests by injecting the acid degradation sample of TRL that was heated with 1 N HCl at 80 °C for 30 min. The parameters investigated were number of theoretical plates (N), tailing factor (T), capacity factor (K) and resolution (R_s_), as presented in (Table 1).

**Table 1:** System suitability test of HPLC-UV method for determination of TRL and its acid degradation product.

Parameter	TRL	Acid degradation product	Limits
N	2104	2323	>2000
T	1.01	1.03	≤2
K	1.19	1.83	≥1
R_s_	3.05		>2

^*^N indicates the number of theoretical plates; T, tailing factor; K, capacity factor; R_s_, resolution factor.

## Discussion

The current study offers multifaceted analysis of TRL as guidance to be further used by QC labs according to their preferences. The main rational behind the multifaceted analysis is to provide many varieties for the analyst to overcome the limitations of the different analytical systems as the high cost of LC-MS/MS for the developing countries, low sensitivity of UV spectrophotometry & the time consuming procedures dealing with the LC-UV methods from preparing different mobile phases to complex instrumentation.

### Linearity

Linearity and all the validation parameters were studied according to ICH guidelines^9^. For each analytical method in the current study, a calibration curve was constructed by plotting the corresponding responses of TRL samples against its concentrations. TRL was analyzed by the optimized methods using six calibrators. The regression equations were computed and accepted linearity of all the methods were ascertained by the regression coefficient (r^2^) values, as shown in (Table 2). Acceptable linearity ranges of TRL were 50–800 ng/mL, 2.5–80 μg/mL, 5–100 μg/mL, 5–100 μg/mL and 5–50 μg/mL for methods I-V, respectively.

**Table 2:** Results obtained by the proposed methods for the determination of TRL.

Item	UPLC-MS/MS	UPLC-UV	HPLC-UV	UHPLC-UV	Direct UV
Linearity range	50–800 ng/mL	2.5–80 μg/mL	5–100 μg/mL	5–100 μg/mL	5–50 μg/mL
Regression equation	A = 0.0051 C ng/mL + 0.0213	A = 0.5084 C μg/mL + 0.1923	A = 0.4738 C μg/mL + 0.3337	A = 0.5019 C μg/mL + 0.5494	A = 0.0276 C μg/mL + 0.0056
Regression coefficient (r^2^)	0.9998	0.9999	0.9999	0.9999	0.9998
LOD	13.52 ng/mL	0.79 μg/mL	0.95 μg/mL	1.12 μg/mL	1.01 μg/mL
LOQ	40.98 ng/mL	2.40 μg/mL	2.89 μg/mL	3.40 μg/mL	3.05 μg/mL
*S_b_	3.12 × 10^-5^	1.84 × 10^-3^	1.70 × 10^-3^	2.11 × 10^-3^	2.16 × 10^-4^
*S_a_	0.017	0.085	0.126	0.157	0.008
Confidence limit of the slope	0.0051 ± 1.58 × 10^−7^	0.5084 ± 9.36 × 10^−4^	0.4738 ± 8.04 × 10^−4^	0.5019 ± 1.06 × 10^−3^	0.0276 ± 5.95 × 10^−6^
Confidence limit of the intercept	0.0213 ± 3.66 × 10^−4^	0.1923 ± 1.64 × 10^−2^	0.3337 ± 4.20 × 10^−2^	0.5494 ± 8.61 × 10^−2^	0.0056 ± 4.50 × 10^−5^
Standard error of the estimation	0.02	0.12	0.14	0.17	0.01
Accuracy (Mean ±S.D.)	99.90 ± 1.54	100.81 ± 1.06	101.09 ± 1.47	100.24 ± 1.46	100.20 ± 1.07
Precision Intraday %R.S.D	1.35–0.56–0.88	0.56–1.68–1.04	0.81–1.02–1.10	1.94–0.34–0.63	1.08 - 0.80 - 0.98
Drug in dosage form, (Mean ± S.D.)	100.05 ± 1.64	100.14 ± 1.57	100.85 ± 1.15	100.58 ± 1.26	99.32 ± 1.11

### Accuracy

Accuracy was determined by calculating % recoveries of TRL different five concentrations for all proposed techniques. All the results including mean of the % recovery and standard deviations for each method are shown in (Table 2). In the sake of confirming the accuracy of the optimized HPLC-UV (the analysis method of choice in TRL kinetic degradation study), standard addition technique was applied and the TRL concentrations were calculated using the corresponding regression equation as in (Table 2).

### Precision

Precision of the proposed methods was confirmed and the % RSD results were < 2% for all the optimized methods, as shown in (Table 2). The % RSD of interday precision for the optimized HPLC-UV method were (0.60 - 0.69–1.01).

### Specificity

**Figure 7:**
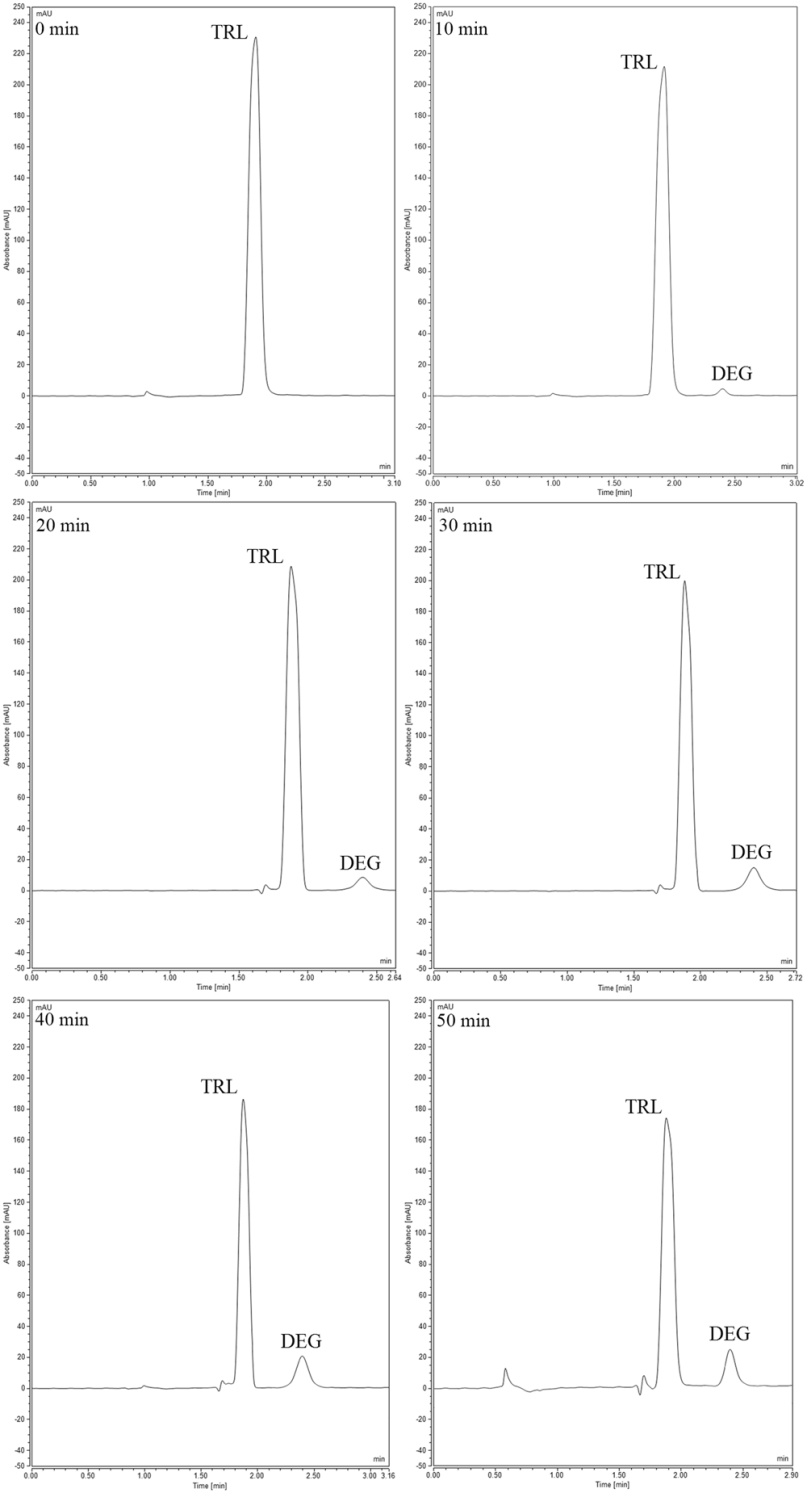
HPLC acid degradation chromatograms of TRL and its main degradation product (DEG) withdrawn at 0, 10, 20, 30, 40 and 50 min at 70 °C.

**Figure 8:**
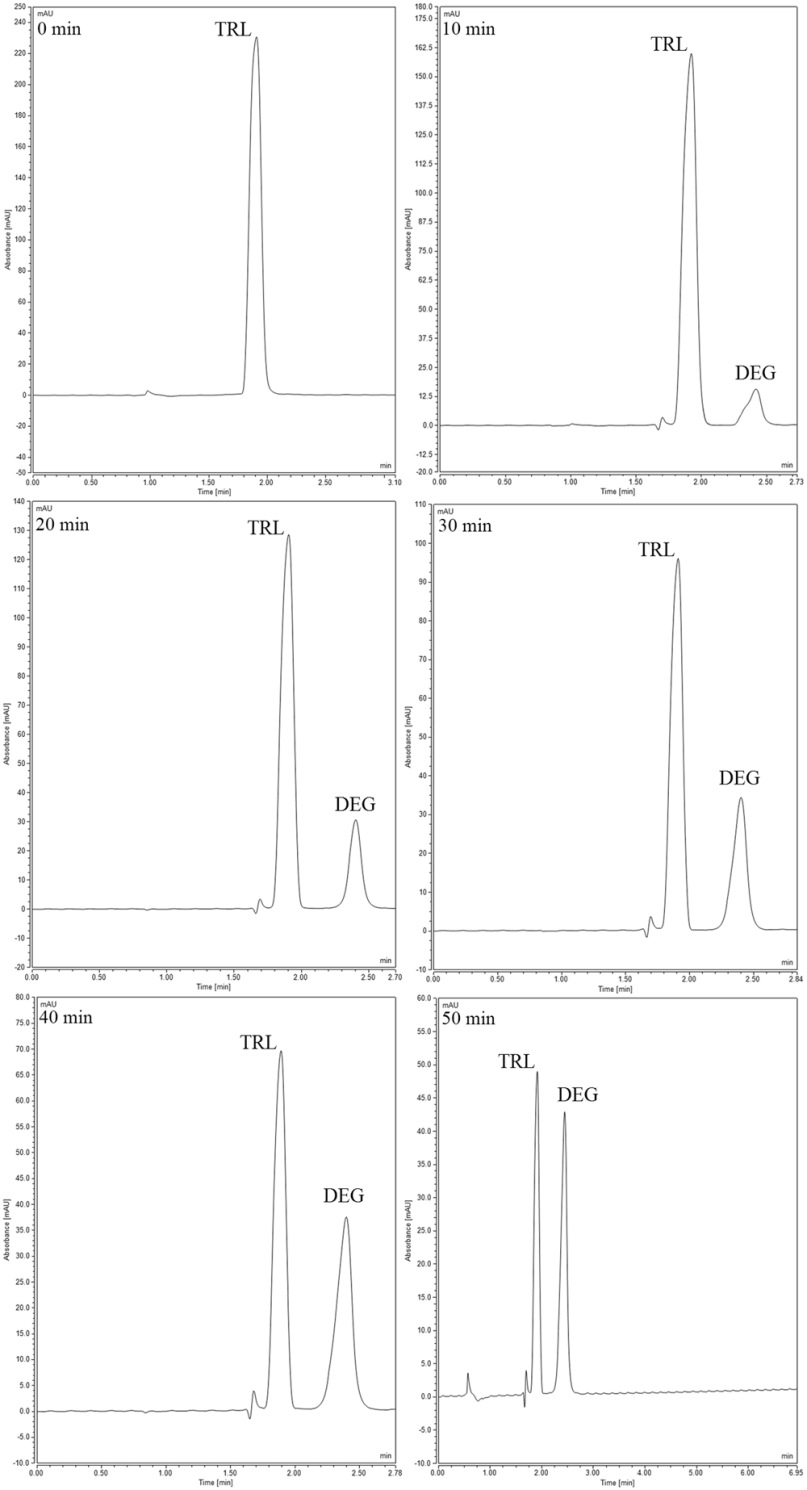
HPLC acid degradation chromatograms of TRL and its main degradation product (DEG) withdrawn at 0, 10, 20, 30, 40 and 50 min at 80 °C.

**Figure 9:**
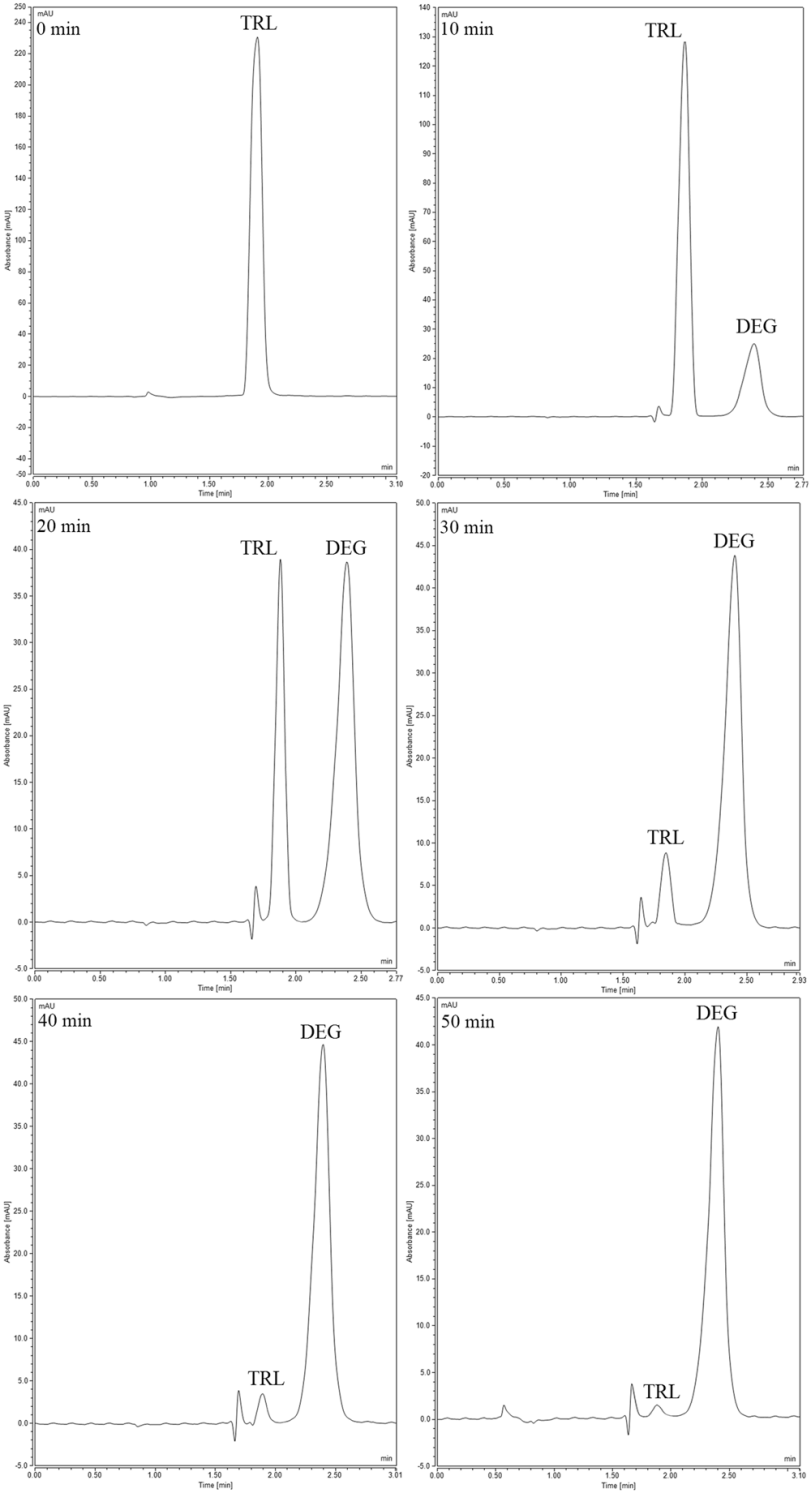
HPLC acid degradation chromatograms of TRL and its main degradation product (DEG) withdrawn at 0, 10, 20, 30, 40 and 50 min at 90 °C.

Specificity of the proposed methods was investigated through determining three concentrations of TRL in the presence of its dosage form excipients. The values of the mean of percent recoveries and the standard deviations for each method are presented in (Table 2). The specificity of the optimized HPLC-UV method was tested by the assay of different acid degradation samples of TRL with high concentrations of the degradation product, as shown in chromatograms of (Figs 7, 8 and 9). Findings were satisfactory to prove that the optimized HPLC-UV method is specific for the drug analysis in presence of up to 98% of its degradation products.

### Pharmaceutical dosage forms and standard addition technique

The proposed methods were successfully used for the determination of TRL in its pharmaceutical dosage form. Standard addition technique was applied for the HPLC-UV method and the TRL concentrations were calculated using the corresponding regression equations as in (Table 2). The resultant mean of recovery % ± standard deviation was equal 100.21 ± 0.95.

### Limit of detection and limit of quantification

Limit of detection (LOD) and limit of quantitation (LOQ) were calculated for each of the proposed methods and its results are demonstrated in (Table 2).

### Robustness

Small variations in the chromatographic conditions of the HPLC-UV method revealed no significant difference, as shown in (Table 3), concluding that the optimized HPLC-UV method was of good robustness.

**Table 3:** Results of HPLC-UV robustness for TRL 50 µg/mL.

Parameter	Optimized method	Flow rate ± 0.02 (mL min^−1^)		pH ± 0.1		Mobile phase composition ± 2% (Acetonitrile: Phosphate buffer)		Column oven temperature ± 2 °C	
		0.48	0.52	3.4	3.6	48%: 52%	52%: 48%	23 °C	27 °C
Area under the peak, Recovery %	101.98	99.66	99.56	100.64	100.59	101.66	100.22	100.15	100.58
Retention time, min	1.86	1.84	1.85	1.89	1.85	1.87	1.86	1.88	1.85
Peak height, mAU	235.76	238.55	241.21	233.07	237.23	238.34	234.39	239.44	240.8

### Statistical Comparison

The optimized methods were compared statistically using one-way analysis of variance (ANOVA) for TRL in raw material & in its pharmaceutical dosage form as shown in (Table 4).

**Table 4:** Statistical comparison between the proposed techniques for the determination of TRL in raw material and in its pharmaceutical dosage form.

	TRL in raw material					TRL in Pharmaceutical dosage form				
	UPLC-MS/MS	UPLC-UV	HPLC-UV	UHPLC -UV	Direct UV	UPLC-MS/MS	UPLC-UV	HPLC-UV	UHPLC -UV	Direct UV
Mean	99.90	100.81	101.09	100.24	100.2	100.05	100.14	100.85	100.58	99.32
±S.D.	1.54	1.06	1.47	1.46	1.07	1.64	1.57	1.15	1.26	1.11
%RSD	1.54	1.05	1.45	1.46	1.07	1.63	1.57	1.14	1.25	1.12
N	5	5	5	5	5	3	3	3	3	3
V	2.37	1.12	2.16	2.13	1.15	2.67	2.46	1.32	1.59	1.24

^*^ANOVA results at *p = *0.05 confirmed that there is no significant difference between groups of TRL in raw material (F = 0.663 & P = 0.625) and in its pharmaceutical dosage form (F = 0.551 & P = 0.703).

### Stability-indicating study

As reported in the literature, one main acid degradation product of TRL (DEG), (2-[(3-methyl-2, 4, 6-oxo-tetrahydro-pyrimidin-1(2 H)yl)-methyl]-4-fluorobenzonitrile), was found at m/z 275.07^5,6^. Literature results were ascertained by separating TRL from its acid degradation product on the TLC that results in two separated spots. The best chromatographic separation of TRL from its main degradation product was obtained by the optimized HPLC-UV method. The retention times for TRL and its acid degradation product were 1.86 and 2.43, respectively. In addition to the current degradation kinetic study, TRL was subjected to preliminary stressed degradation conditions including alkaline, 0.3% H_2_O_2_, UV light and heat stress conditions. Among all the stressed conditions, TRL was mostly sensitive to acidic conditions that are comparable with the literature results of ALO, as shown in (Table 5).

**Table 5:** Summary of preliminary investigation of TRL forced degradation.

Stress condition	Time	Remaining Trelagliptin, %
Acid hydrolysis (1.0 N HCl, 80 °C)	0.5 h	44.55
Base hydrolysis (1.0 N NaOH, 80 °C)	2.0 h	81.41
Oxidation (0.3% H_2_O_2_)	2.0 h	98.86
Thermal (80 °C)	2.0 h	99.72
UV light (4500 lx)	2 days	99.59

### Kinetic degradation study

**Figure 10:**
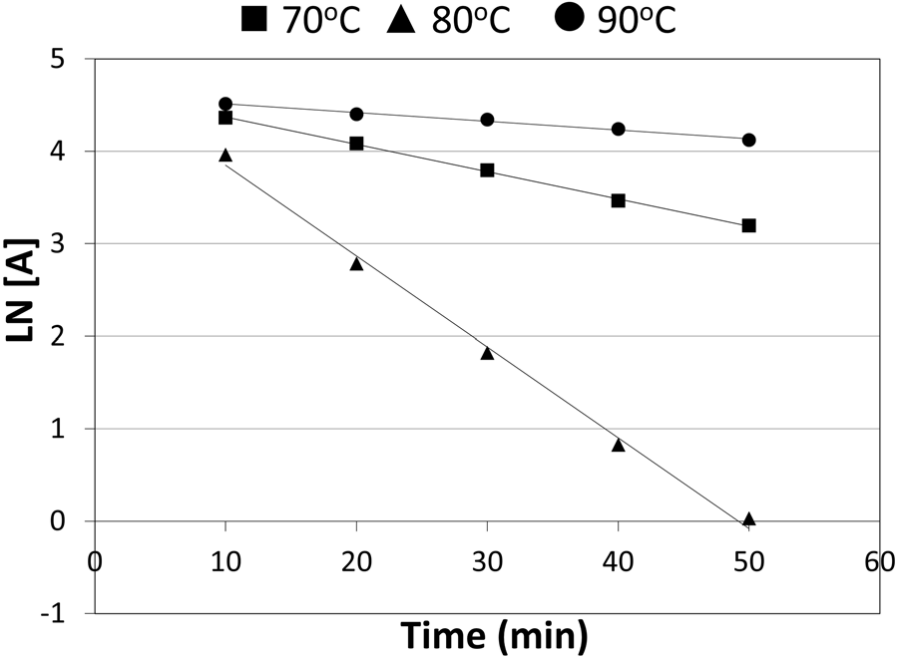
First order kinetic plot of the acid degradation of TRL at 70 °C, 80 °C and 90 °C showing kinetic plots of the natural logarithm (Ln) of the TRL remaining percent versus time for each temperature.

**Figure 11:**
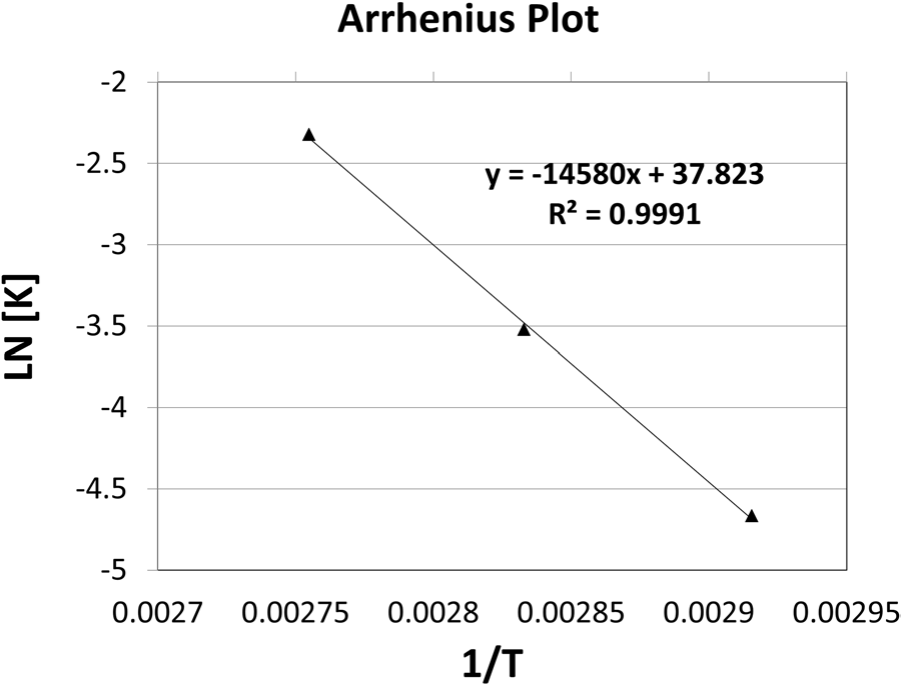
Arrhenius plot of the acid degradation of TRL showing the natural logarithm of (K) values for each temperature against the corresponding (1/T) inverse absolute temperature.

The linear acid degradation results were satisfactory to apply the current kinetic degradation study. TRL kinetics degradation study was performed in 1 N HCl. The increase of the heating time intervals with acid led to decrease in the concentration of TRL and further increase in its main degradation product concentration. The effect of changing temperature was investigated through different temperatures (70 °C, 80 °C and 90 °C), as shown in (Figs 7, 8 and 9, respectively). The degradation samples were analyzed through the optimized HPLC-UV method. Kinetic plots of the natural logarithm (Ln) of the TRL remaining percent versus time, in minutes, for each temperature were constructed. The resultant charts were obeyed the first-order degradation kinetics (Fig. 10). Kinetic plot findings, the regression equation for each kinetic plot is following this equation (Equation 1):

1 $$\mathrm{Ln}\,({\rm{T}})=\,\mathrm{Ln}\,({{\rm{T}}}_{{\rm{o}}})\,\mbox{--}\,{\rm{Kt}}$$

where, Ln (T) refers to the natural logarithm of TRL remaining percent, Ln (T_o_) refers to the natural logarithm of TRL initial concentration percent, (K) refers to the rate constant of the apparent first order degradation, (t) refers to the temperature at which the degradation took place. Then, calculating the half-life (t_1/2_) which equals to 0.693 divided by (K) and the shelf-life (t_90_) which equals to 0.10536 divided by (K) for each temperature. Then, the Arrhenius plot was constructed by plotting the natural logarithm of (K) values for each temperature against the corresponding (1/T) inverse absolute temperature, (Fig. 11). After that, calculating K at 25 °C from the following Arrhenius plot regression equation (Equation 2) was performed:

2 $$\mathrm{Ln}\,({\rm{K}})=\,\mathrm{Ln}\,({\rm{A}})-{\rm{Ea}}/({\rm{R}}\ast {\rm{T}}),$$

where, Ln (K) refers to the natural logarithm of the apparent first order degradation rate constant at certain temperature, Ln (A) refers to the natural logarithm of the Arrhenius frequency factor (which is the intercept in the regression equation), (Ea) refers to the activation energy, (R) refers to the gas constant (which is a constant value that equals 1.987 cal/k^*^mol), (Ea / R) refers to the slope in this regression equation and (T) refers to the absolute temperature in Kelvin at which the degradation took place. Then calculating the t_1/2_ and t_90_ at 25 °C was carried out. Finally calculating the Ea which equals to the slope value in the Arrhenius plot regression equation * R was accomplished. The conclusion from the current kinetic study is that the increment of the temperature conversely increase the rate of degradation and inversely decrease the t_1/2_, as demonstrated in the kinetic study results in (Table 6).

**Table 6:** Summary of degradation kinetics study of TRL in 1 N HCL.

Temperature (°C)	K (min^−1^)	t_1/2_ (min)	t_90_ (min)	E_a_ (K.cal.mol^−1^)	A (min^−1^)
70	9.4 × 10^−3^	73.5	11.2		
80	3.0 × 10^−2^	23.4	3.6		
90	9.8 × 10^−2^	7.1	1.1		
Value at 25 °C	1.5 × 10^−5^	46002.0	6993.9	28.97	2.67 × 10^16^

^*^Where, K is the degradation rate constant, t_1/2_ is the half life, t_90_ is the shelf life, Ea is the activation energy and A is the Arrhenius frequency factor.

## Conclusion

Accuracy and reproducibility of the multifaceted analysis were confirmed for TRL quantification. The proposed methods validity was confirmed by its satisfactory results for all the parameters tested and they are suitable methods to be used by quality control laboratories. The UPLC-UV method had a lower quantification limit from 2.5 to 80 µg/mL. While the HPLC-UV and UHPLC-UV methods had higher linearity range with concentrations from 5 to 100 µg/mL. Direct UV method was confirmed to be an economic analytical method for TRL quantification, but with a lower linearity range of 5–50 µg/mL. TRL was mostly sensitive to acidic conditions although its minimal responses to the other stressed conditions. The HPLC-UV was a highly sensitive method with good resolution and robustness for the analysis of TRL and its acid degradation product. Arrhenius plot was used in the kinetic study and the apparent 1^st^ order degradation rate constant (K), t_1/2_, t_90_, and the activation energies were calculated for each temperature and at 25 °C. The UPLC-MS/MS method was of the highest sensitivity for the TRL analysis (in a nano gram scale), that supports its further use for TRL assay in the biological fluids.
